# mu-Opioid Receptor Polymorphisms and Breast Cancer Recurrence in Adult Korean Women Undergoing Breast Cancer Surgery: A Retrospective Study

**DOI:** 10.7150/ijms.49297

**Published:** 2020-10-18

**Authors:** Yea-Ji Lee, Chung-Sik Oh, Ji Min Choi, Sangtae Park, Seong-Hyop Kim

**Affiliations:** 1 Department of Anaesthesiology and Pain medicine, Konkuk University Medical Centre, Konkuk University School of Medicine, Seoul, Korea; 2 Research Institute of Medical Science, Konkuk University School of Medicine, Seoul, Korea; 3 Department of Infection and Immunology, Konkuk University School of Medicine, Seoul, Korea

**Keywords:** Receptors, opioid, mu, Breast neoplasms, Anaesthesia, general

## Abstract

**Background:** Genetic variations of mu-opioid receptors are well known to contribute to growth and progression of tumors. The most common single-nucleotide polymorphism (SNP) in the mu-opioid receptor 1 gene (OPRM1) is the A118G mutation. We examined the association between the recurrent breast cancer and genotypes of *OPRM1* A118G SNP (AA vs. AG vs. GG) in Korean women population.

**Methods:** We analysed medical records and genetic data of 200 patients aged more than 20 who underwent primary breast cancer surgery from June 2012 to June 2014 and diagnosed recurrent breast cancer from June 2012 to September 2019.

**Results:** The incidence of recurrent breast cancer was 6.1%, 8.2%, and 4.8% in genotype AA, AG and GG, respectively (*p*=0.780). The incidence of recurrent breast cancer in volatile anaesthesia group was 7.0% and 7.1% in total intravenous anaesthesia (TIVA) group (RR = 0.984, 95% CI = 0.328 - 2.951; *p* = 0.978).

**Conclusion**: *OPRM1* A118G SNP had no influence on breast cancer recurrence in Korean women. Anaesthesia technique did not show significant effect on the incidence of recurrent breast cancer.

## Introduction

Opioid receptors are involved in tumour growth and progression; however, the mechanisms underlying the pro-tumourigenic effects of opioid receptors are not fully understood 1-3. As the role of genetic variation in tumour occurrence and progression has become evident, the potential tumour-promoting effects of single-nucleotide polymorphisms (SNPs) in opioid receptors have been assessed in various tumour entities 4-5. The most common SNP in the mu opioid receptor gene (*OPRM1*) is the adenine to guanine substitution (A118G) 6-7. Efforts to determine the clinical relevance of *OPRM1* A118G SNP in breast cancer have yielded conflicting findings 6-8 limiting its potential use as a prognostic marker in breast cancer patients.

The aim of this retrospective study was to assess the potential link between *OPRM1* A118G SNP and breast cancer recurrence. We investigated the impact of *OPRM1* A118G SNP on breast cancer recurrence in Korean adult female patients. We also evaluated the effect of anaesthetic technique on breast cancer recurrence in the same population.

## Patients and Methods

### Study population and medical record review

After protocol review and approval by the Institutional Review Board of Konkuk University Medical Centre, Seoul, South Korea (KUH2020-04-039), data from adult Korean women who had undergone breast cancer surgery under general anaesthesia at Konkuk University Medical Centre from June 2012 to June 2014 were retrospectively reviewed. Before breast cancer surgery, the patients were examined to confirm that the primary origin of the lesion was the breast. Breast cancer was also confirmed by pathological examination of biopsies taken during surgery. Data regarding pathological findings, cancer stage, anaesthetic technique during surgery, and postoperative cancer treatment were reviewed. Information regarding breast cancer recurrence within 5 years (from June 2012 to June 2019) was acquired from the medical records, and cases with tumour recurrence were classified into different categories, as previously described 9. Local recurrence was defined as tumour recurrence in the breast where the cancer was originally diagnosed, or in the skin or subcutaneous tissue of the ipsilateral chest wall. Regional recurrence was defined as recurrence in ipsilateral axillary lymph nodes or the collarbone area. Distant recurrence was defined as recurrence in another part of the body, such as the lungs, bones, brain, or contralateral breast. The pathological findings of recurrent breast tumours were also reviewed. Patients diagnosed with benign lesions, as well as those who were diagnosed with other primary cancer during the study period, and those lost to follow-up, were excluded from the study.

### Genotyping assays

*OPRM1* A118G SNP genotype data were obtained from our previous study; the procedure followed for *OPRM1* genotyping has also been previously described 4,10.

### Anaesthesia technique

Information regarding the anaesthetic technique used during surgery was obtained from the medical records 4,10. Patients received either volatile anaesthesia or total intravenous anaesthesia (TIVA). None of the patients received pre-anaesthetic medications. After routine patient monitoring, anaesthesia was induced. Lidocaine, at a dose of 0.5 mg/kg, was used to reduce pain on propofol injection. In patients that received volatile anaesthesia, anaesthesia was initiated with bolus injection of propofol (2 mg·kg^-1^) and maintained with sevoflurane, as well as target-controlled infusion (TCI) of remifentanil. In patients who underwent TIVA, anaesthesia was induced and maintained using effect-site and plasma TCI of propofol and remifentanil, respectively. A target plasma remifentanil concentration of 10 ng·mL^-1^ was maintained during surgery in all patients in both groups. The end-tidal concentration of sevoflurane and target concentration of propofol at the effect site were titrated to maintain bispectral index values between 40 to 60. Rocuronium was administered for muscle relaxation and neuromuscular paralysis was antagonized with neostigmine and glycopyrrolate under monitoring with peripheral nerve stimulator.

### Statistics

Breast cancer recurrence was analysed according to the *OPRM1* A118G SNP (AA *vs.* AG *vs.* GG genotype). Breast cancer recurrence was also compared according to the anaesthetic technique applied during breast cancer surgery (volatile anaesthesia *vs.* TIVA). The sample size for these analyses was based on data availability.

Statistical analyses were performed using SPSS software (ver. 25.0; IBM Corp., Armonk, NY, USA). The normality of the data was tested using the Shapiro-Wilk test (results not shown). Student's *t*-test was used to compare normally distributed data, while the Mann-Whitney *U* test was used to compare non-normally distributed variables. One-way ANOVA test or Kruskal-Wallis test was used to analyse categorical data. Data are expressed as number of patients (%), means ± standard deviation (SD) or median [range]. *P*-values < 0.05 were considered significant. Relative risks (RR) and 95% confidence intervals (CI) were calculated using chi-square test for the incidence of recurrent breast cancer.

## Results

For this study, we used data from our previous study involving 408 breast cancer patients 4. However, 158 patients with benign disease, 8 with other primary cancer (4 with thyroid cancer, 1 with lung cancer, 1 with colon cancer, 1 with endometrial cancer, and 1 with cervical cancer), and 42 who were lost to follow-up were excluded. Therefore, data from 200 patients were analysed in the present study.

**Figure 1:**
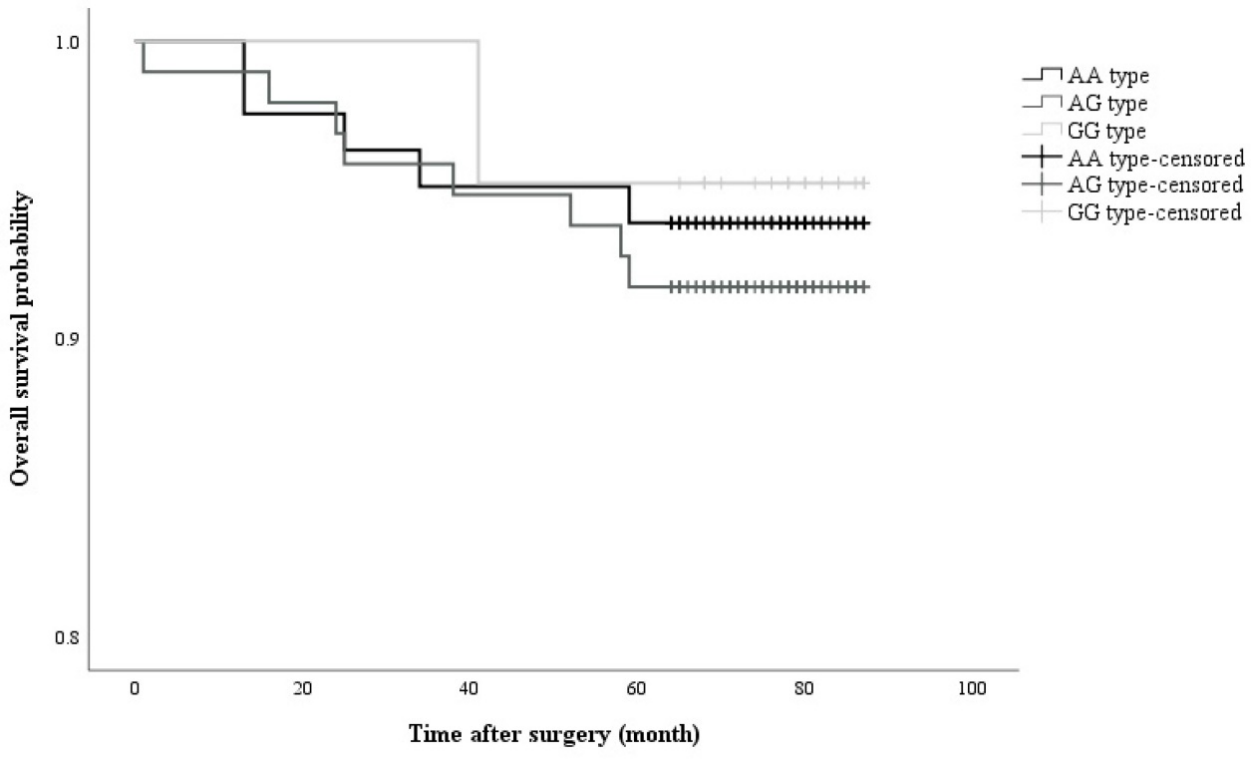
Kaplan-Meier curve showing cumulative survival of recurrent cancer in accordance with opioid receptor polymorphism (Log rank P value = 0.782)

Analysis of the *OPRM1* A118G SNP revealed that 40.7% of patients had the AA genotype, 49.0% the AG genotype, and 10.3% the GG genotype. No significant differences in patient demographics were observed among patients with different *OPRM1* A118G SNPs (Table 1). Tumour recurrence after breast cancer surgery occurred in 14 out of 200 patients (7.0%) and the incidence of recurrence did not differ significantly among the groups (Table 1). Consistently, the characteristics of the patients with recurrent breast cancer were similar among the different groups (Table 2) (Figure 1).

**Figure 2:**
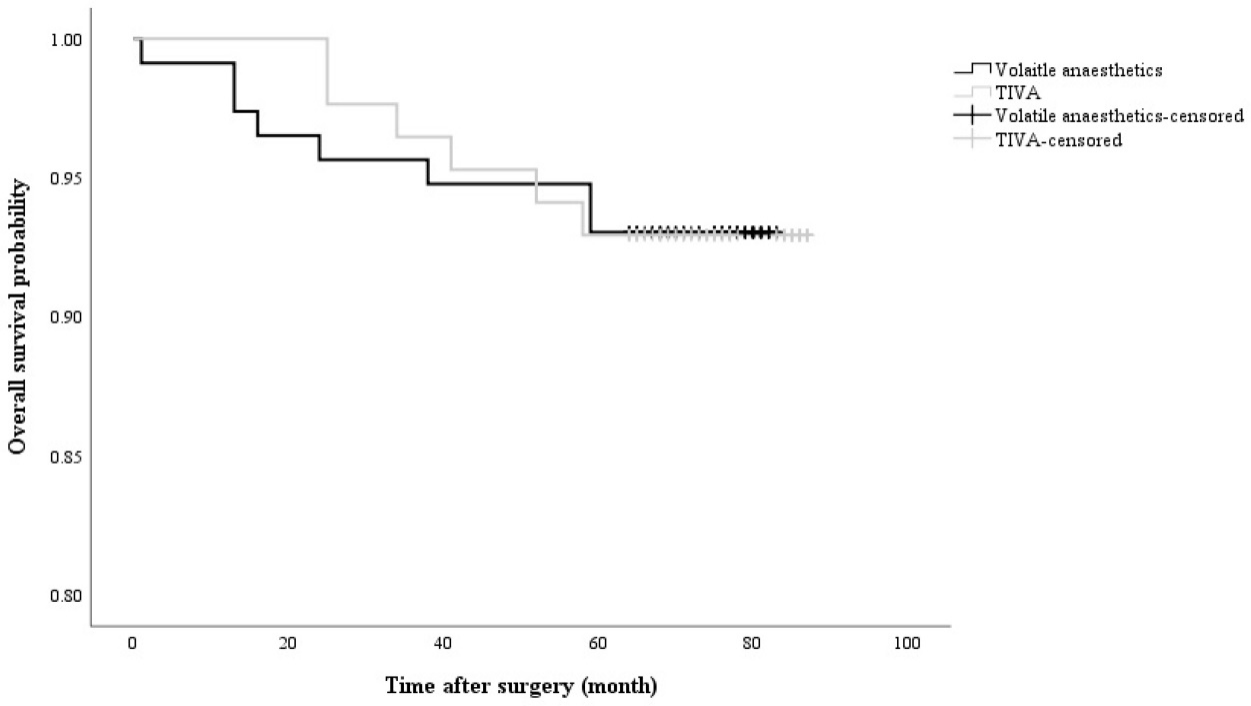
Kaplan-Meier curve showing cumulative survival of recurrent cancer in accordance with anaesthetic agents (Log rank *p* value = 0.997). TIVA: total intravenous anaesthesia

The presence of the G allele, which was not significantly associated with breast cancer recurrence, was observed in 7.6% of patients with breast cancer recurrence, while 6.1% of patients with recurrence did not have the G allele (RR = 1.272, 95% CI = 0.410 - 3.942; *p* = 0.677). Moreover, the anaesthetic technique did not significantly affect breast cancer recurrence (volatile anaesthesia in 7.0% of recurrent patients *vs.* TIVA in 7.1%; RR = 0.984, 95% CI = 0.328 - 2.951; *p* = 0.978) (Figure 2). No significant differences in demographics between the volatile anaesthesia group and the TIVA group (Table 3).

## Discussion

In this study, *OPRM1* A118G SNP was not associated with breast cancer recurrence in Korean adult women after breast cancer surgery; furthermore, the presence of the G allele did not affect tumour recurrence. The incidence of tumour recurrence after surgery was similar between the patients who received volatile anaesthesia and those who underwent TIVA during surgery, suggesting that there was no link between anaesthetic technique and tumour recurrence in this cohort.

Opioid receptor activation in endothelial cells promotes angiogenesis and immune suppression 11-13. Therefore, opioid receptors can affect tumour growth and progression. *OPRM1* A118G SNPs, in particular the GG genotype, have been linked to the development of resistance to exogenous opioids, and patients harbouring the GG genotype require higher doses of exogenous opioids to manage postoperative pain 14-16. Additionally, *OPRM1* A118G SNPs have been reported to affect tumour growth and progression in breast cancer patients. Bortsov et al. 6 reported that American breast cancer patients with one or more copies of the G allele exhibited improved survival outcomes. They also showed that the frequency of G allele was lower in patients with advanced-stage breast cancer. On the contrary, a study of Polish breast cancer patients by Cieślińska et al. 8 suggested a positive association between the AG and GG genotypes and the incidence of breast cancer in north-eastern European Caucasians. They therefore concluded that the presence of the G allele was a significant risk factor for breast cancer development. However, Oh et al. 4 failed to confirm an association between the AG and GG genotypes and the incidence of breast cancer in Korean patients. They concluded that the conflicting results regarding the relationship between *OPRM1* A118G SNPs and breast cancer incidence could be explained by the multifactorial nature of breast cancer initiation and progression, both of which are also influenced by environmental factors.

Differences in the genetic makeup of the cohorts among different studies might also contribute to contradictory results. For example, the relevance of leptin signalling in breast cancer development differs according to the ethnicity of patients. Although alterations in leptin signalling have been associated with a significantly elevated risk of breast cancer development in Korean cohorts, no link between leptin receptor (LEPR) Gln223Arg polymorphism and breast cancer development was observed in a European cohort 17.

In the present study, we also assessed the role of the anaesthetic technique used during breast cancer surgery in the incidence of breast cancer recurrence. Numerous studies showed that intravenous administration of anaesthetic agents provided more favourable outcomes compared with volatile anaesthetic agents 18-21. However, these results should be reconsidered whether the same hypnotic effect of intravenous and volatile anaesthetic agents was compared. For the clearer comparison of the same hypnotic effect of intravenous and volatile anaesthetic agents, we applied anaesthetic depth monitoring and fixed the target concentration of remifentanil in both groups. In contrast to previous studies, we found that the anaesthetic agents had no influence on breast cancer recurrence. Future studies are required to investigate whether this is also the case when different concentrations of remifentanil are used.

Our study had several limitations. Firstly, the effect of the G allele on intraoperative haemodynamic parameters and postoperative pain should be assessed to confirm the relevance of *OPRM1* A118G SNPs to breast cancer recurrence. Secondly, the study was retrospective in nature, potentially leading to bias due to the presence of confounding factors. Even though the genotype data and information for anaesthetic technique were obtained from our previous study, a prospective double-blinded randomized clinical trial 10, the selection bias was not completely avoidable. Thirdly, the number of recurrent cancer patients for each genotype group was too small. For obtaining more reliable results, the large population study should be conducted in the future.

In conclusion, neither the presence of *OPRM1* A118G SNPs nor the anaesthetic technique used during breast cancer surgery had an effect on breast cancer recurrence in Korean adult patients.

## Figures and Tables

**Table 1:** Demographic data in accordance with opioid receptor polymorphism

	AA (*n* = 82)	AG (*n*=97)	GG (n=21)	*p* value
Age (year)	48.8 ± 9.3	48.6±9.2	53.2±10.6	0.112
Height (cm)	157.5±6.9	157.6±5.8	157.6±5.0	0.977
Weight [kg]	55.4 [51.8-62.5]	57.5 [51.7-62.4]	56.9 [53.9-60.7]	0.840
Final pathologic results				0.729
**DCIS**	14 (17.1)	20 (20.6)	5 (23.8)	
** IBC**	68 (82.9)	77 (79.4)	16 (76.2)	
Cancer Stage				0.729
**Stage 0**	0 (0.0)	1 (1.0)	0 (0.0)	
**Stage** I	55 (67.1)	56 (57.7)	15 (71.4)	
** Stage** II	14 (17.1)	23 (23.7)	4 (19.0)	
** Stage** III, IV	13 (15.9)	17 (17.5)	2 (9.5)	
Anaesthesia time (min)	124.4±36.4	122.8±46.9	132.7±40.6	0.620
Operation time (min)	91.4±33.9	90.4±45.6	96.9±43.0	0.804
Sevoflurane [vol%] **Minimum**	0.7 [0.0-1.0]	0.8 [0.0-1.0]	0.0 [0.0-1.0]	0.273
**Maximum**	1.2 [0.0-1.5]	1.3 [0.0-1.7]	0.0 [0.0-1.6]	0.206
Propofol [mg]	141.9 [111.3-546.3]	128.0 [109.4-450.5]	277.0 [116.1-635]	0.153
Remifentanil (μg)	2556.7.0±815.6	2581.8±1083.3	2623.1±1007.3	0.959
Postoperative treatment				
**CTx.**	48 (58.5)	59 (60.8)	11 (52.4)	0.771
**RTx.**	77 (93.9)	89 (91.8)	20 (95.2)	0.780
**HTx.**	72 (87.8)	82 (84.5)	20 (95.2)	0.401
Incidence or recurrence	5 (6.1)	8 (8.2)	1 (4.8)	0.780

Values are expressed as mean ± SD, median [range] or number of patients (%). DCIS: ductal carcinoma in situ; IBC: invasive breast cancer; CTx.: chemotherapy; RTx.: radiation therapy; HTx.: hormonal therapy.

**Table 2:** Recurrent cancer patient diagnosis

	AA (*n*=5)	AG (*n*=8)	GG (n=1)	*p* value
Treatment^*^				
CTx.	1 (14.3)	5 (71.4)	1 (14.3)	0.192
RTx.	4 (33.3)	7 (58.3)	1 (8.3)	0.852
**HTx.**	4 (33.3)	7 (58.3)	1 (8.3)	0.852
Type of recurrence				0.297
Local recurrence	4 (80.0)	2 (25.0)	1 (100)	
Regional recurrence	0	1 (12.5)	0	
Distant recurrence	1 (20.0)	5 (62.5)	0	
Pathologic results^†^				0.607
DCIS	2 (40.0)	2 (25.0)	0	
IBC	2 (40.0)	2 (25.0)	1 (100)	
Other breast cancer	1 (20.0)	1 (12.5)	0	
Distant metastasis	0	3 (37.5)	0	

Values are number of patients (%). ^*^ Treatments were performed after breast cancer surgery. ^†^Pathologic results were confirmed by surgical biopsy after recurrent breast cancer was detected. DCIS: ductal carcinoma in situ; IBC: invasive breast cancer; CTx.: chemotherapy; RTx.: radiation therapy; HTx.: hormonal therapy.

**Table 3:** Demographic data in accordance with anaesthetic technique

	Volatile Anesthesia Group (*n*=115)	TIVA Group (n=85)	*p* value
Polymorphism (AA/AG/GG)	45/60/10 (39.1/52.2/8.7)	37/37/11 (43.5/43.5/12.9)	0.402
Age [year]	48 [43-55]	48 [43-55]	0.830
Height (cm)	157.9±5.9	157.1±6.5	0.400
Weight [kg]	57.5 [52.5-63.0]	55.6 [50.6-61.5]	0.271
Anaesthesia time [min]	116 [95-145]	126 [104-155]	0.037^*^
Operation time [min]	87 [65-111]	88 [67-118]	0.411
Sevoflurane [vol%]			
Minimum	1.0 [0.9-1.1]		
Maximum	1.5 [1.4-1.8]		
Propofol [mg]	114.4 [104.0-126.2]	540 [419.0-699.0]	<0.001^*^
Remifentanil [mcg]	2396.0 [1872.0-3046.0]	2696.0 [1954.0-3332.5]	0.157
Incidence of recurrence	8 (7.0)	6 (7.1)	0.978

Values are number of patients (%), median [range] or mean ± SD. *: *p* <0.05
